# Preferring more e-cigarette flavors is associated with e-cigarette use frequency among adolescents but not adults

**DOI:** 10.1371/journal.pone.0189015

**Published:** 2018-01-04

**Authors:** Meghan E. Morean, Ellyn R. Butler, Krysten W. Bold, Grace Kong, Deepa R. Camenga, Dana A. Cavallo, Patricia Simon, Stephanie S. O’Malley, Suchitra Krishnan-Sarin

**Affiliations:** 1 Department of Psychology, Oberlin College, Oberlin, OH, United States of America; 2 Department of Psychiatry, Yale School of Medicine, New Haven, CT, United States of America; 3 Department of Emergency Medicine, Yale School of Medicine, New Haven, CT, United States of America; Louisiana State University, UNITED STATES

## Abstract

**Introduction:**

Many e-cigarette users find the variety of e-cigarette flavors appealing. We examined whether preferences for e-liquid flavors and the total number of flavors preferred differed between samples of adolescent and adult e-cigarette users. We also examined whether these preferences were associated with e-cigarette use frequency for adolescents or adults, respectively.

**Materials and methods:**

The analytic samples comprised 1) 396 adolescent, past-month e-cigarette users from 5 Connecticut high schools who completed an anonymous, school-based survey in Fall 2014 (56.1% male; 16.18 [1.18] years; 42.2% past-month smokers), and 2) 590 adult, past-month e-cigarette users who completed an anonymous, MTurk survey in Fall 2014 (53.7% male; 34.25 [9.89] years; 51.2% past-month smokers).

**Results:**

Compared to adults, a larger proportion of adolescents preferred fruit, alcohol, and “other”-flavored e-liquids, whereas adults disproportionately preferred tobacco, menthol, mint, coffee, and spice-flavored e-liquids (*p-*values < .05). Adults also preferred a greater total number of flavors compared to adolescents and used e-cigarettes more frequently (*p-*values < .001). Flavor preferences uniquely were associated with frequency of e-cigarette use within the adolescent sample; the total number of flavors preferred was associated with more days of e-cigarette use (η_p_^2^ = 0.04), as were preferences for fruit (η_p_^2^ = 0.02), dessert (η_p_^2^ = 0.02), and alcohol-flavored (η_p_^2^ = 0.02) e-liquids.

**Conclusions:**

Flavor preferences differed between adolescent and adult samples. While youth reported less frequent e-cigarette use overall, their preferences for specific flavors and the total number of flavors preferred were associated with more days of e-cigarette use, indicating that flavor preferences may play an important role in adolescent e-cigarette use.

## Introduction

E-cigarette use is increasingly popular among both American adults and adolescents, with recent estimates indicating that approximately 5% of American adults [1] and 16% of American high school students [2] used an e-cigarette in the past month. Although e-cigarette use is most common among adolescents and adults who also smoke cigarettes, nonsmokers use e-cigarettes as well [1]-[2]. While a number of motivations for using e-cigarettes have been identified, the current study focuses on one of the most controversial features of e-cigarettes: the availability of a wide range of e-liquid flavors.

Based on evidence that flavored tobacco encourages youth cigarette smoking [3], the sale of flavored tobacco cigarettes, with the exception of menthol, was banned in 2009 [4]. However, no such ban exists for e-cigarettes, and there currently are thousands of e-liquid flavors available for sale [5]. Although some research indicates that the availability of diverse e-liquid flavors appeals to adults and may assist them in quitting smoking [6]-[8], there is mounting evidence that the plethora of available flavors disproportionately attracts youth to the e-cigarette market. Research indicates that the appeal of e-liquid flavors is linked to youth e-cigarette experimentation/initiation [9], that the vast majority of adolescent e-cigarette users report that they initiated use with an e-cigarette flavored to taste like something other than tobacco [10], and recent national survey data indicate that 81.5% of adolescent e-cigarette users report that flavors are the leading reason for their continued e-cigarette use [11].

Although the use of flavored e-liquids has been linked to e-cigarette use in adolescents and adults, the relationship between the preferences for specific flavors and the frequency of e-cigarette use is not well understood. For example, while prior research indicates that both adult and adolescent e-cigarette users often like to use more than one e-liquid flavor [7] [9], it is not clear if using multiple e-liquid flavors impacts e-cigarette use frequency. It is possible that e-cigarette users may use multiple flavors interchangeably without increasing their overall e-cigarette use. However, preferring to use multiple e-liquid flavors also may lead to increased e-cigarette use. For example, using multiple flavors may help to maintain the novelty of e-cigarette use by increasing choice and, the use of sweet flavors, in particular, may increase palatability by cutting the harshness of nicotine [12].

To address the aforementioned gaps in the research literature, the current study examined potential differences in adolescents’ and adults’ preferences for 10 e-liquid flavors (i.e., tobacco, menthol, mint, fruit, coffee, vanilla, dessert/candy, spices, alcohol, and other) and the total number of flavors preferred by each group. We also examined whether adolescents’ and/or adults’ preferences for individual flavors or the total number of flavors preferred, respectively, were associated with the number of days of e-cigarette use in the past month above and beyond covariates that previously have been shown to predict e-cigarette use frequency (i.e., sex, age, cigarette smoking, and the use of e-liquid containing nicotine) [13].

## Materials and methods

### Participants and procedures

During Fall 2014, two independent cohorts of adolescents and adults were recruited to complete anonymous surveys assessing e-cigarette and other tobacco use. Many of the questions on the surveys overlapped (see Measures section), but the data collection approaches differed for these two cohorts.

#### Adolescents

Prior to administering the school-based, paper-and-pencil survey to adolescents, approval was obtained from the Institutional Review Board of Yale University, the school administrators, and all participating schools. In addition, information sheets were mailed to parents in advance of the study, and parents were instructed to contact the research staff if they did not want their child to participate. No parents withdrew participation for their child. All students were informed that participation was voluntary and that data would be kept confidential. Completing the survey, which occurred during homeroom/advisory periods, served as consent/assent. In total, 4,014 high school students from 5 high schools completed the survey. Ultimately, the analytic sample comprised past-month e-cigarette users who provided answers to the questions assessing e-cigarette use preferences (n = 396 of 472 past-month e-cigarette users; 56.1% male, mean age 16.18 [*SD =* 1.18] years; 42.2% cigarette smokers). Please note that adolescent participants who had missing data represented a higher risk sample than participants with valid data for e-cigarette flavor preferences; participants who had missing data were more likely to be smokers (42.2% vs 27.6%), which previously has been linked to more frequent e-cigarette use [13], and also reported using e-cigarettes more frequently (*M* = 9.98 [SD = 10.52]) days vs. *M* = 6.45 [SD = 9.57] days), which was a central outcome of interest in the current study.

#### Adults

During the same time period, online data were collected for adults through Amazon Mechanical Turk (i.e., Mturk), a crowdsourcing data collection platform that produces valid survey data [14]. The Institutional Review Board of Oberlin College approved the online study. 2,344 individuals provided consent to complete a series of eligibility screener questions. To be eligible, potential participants had to be registered MTurk “Master workers” who had completed at least 5,000 previous MTurk jobs (demonstrating platform familiarity) with an approval rating of at least 95% (demonstrating high quality work); be least 18 years old (mandated of Mturk workers); currently live in the United States; and report past-month e-cigarette use. In total, 627 participants were eligible and provided consent. However, 27 individuals never started the survey and 10 were missing data on e-cigarette flavor preferences. Thus, the analytic sample comprised 590 participants (53.7% male, 69.0% White, mean age 34.25 [*SD =* 9.89] years; 51.2% cigarette smokers). The adult participants with valid data on all study variables did not differ significantly from the 10 participants who had missing data.

### Measures

#### Demographics

All participants reported their sex and age.

#### E-cigarette use frequency

For all participants, past-month e-cigarette use status and frequency were determined using the following question: “During the past 30 days, on how many days did you use an e-cigarette?” (open-ended response).

#### Cigarette smoking status

For adolescents, past-month smoking status was determined using the following question: “During the past 30 days, on how many days did you smoke cigarettes?” Response options included 0, 1, 2, 3–5, 6–10, 11–20, 21–28, and every day. “Current smokers” were defined as adolescents who reported smoking on at least 1 day in the past 30 days. A different question was used to assess current smoking status in adults. For adults, “current smokers” were defined as those who self-reported “yes” to the question ““Did you smoke one or more cigarettes in the past 30 days?”

#### Nicotine content of e-liquid

All participants reported whether they typically “use an e-cigarette with nicotine.” Response options included: “no”, “yes”, “I use e-cigarettes both with and without nicotine”, and “I don’t know.” Individuals who responded “no” were categorized as not using nicotine. Participants who responded “yes” or who indicated that they “use e-cigarettes both with and without nicotine” were categorized as using e-cigarettes with nicotine e-liquid. Note that no participants indicated that they did not know if their e-liquid contained nicotine.

#### E-liquid flavor preferences

Participants reported which e-liquid flavors they preferred to use from the following categories: “tobacco, menthol, mint, fruit (like strawberry, blueberry, or peach), vanilla, candy/dessert (like apple pie, chocolate, or Jolly Rancher), spice (like clove, cinnamon, or nutmeg), alcohol (like piña colada, strawberry daiquiri, or bourbon), coffee (like espresso, latte, or cappuccino)”, “other”, and “I don’t know.” Participants could select as many flavors as were applicable. For all participants, a summary score was created reflecting the total number of flavors preferred (range 0–10). Participants who indicated that they did not know what flavor they preferred or who did not indicate a preference were assigned a score of zero for the total number of flavors preferred.

### Data analytic plan

We conducted statistical analyses using SPSS 24.0 [15]. To explore potential differences between the adolescent and adult samples, chi-squares (for categorical variables) and independent samples t-tests (for continuous variables) were used to examine unadjusted differences in sex, age, smoking status, e-cigarette nicotine content, e-liquid flavor preferences, the total number of e-liquid flavors preferred, and e-cigarette use frequency (i.e., number of days of use in the past 30). We then used univariate general linear modeling to examine potential associations between flavor preferences and e-cigarette use frequency. Models were run separately for adults and adolescents given the differences in the samples and survey methods. The first two models evaluated whether e-cigarette frequency was associated with either adults’ or adolescents’ preferences for each of the ten flavors, which were entered simultaneously into each model. The second two models evaluated whether e-cigarette use frequency was associated with the total number of flavors preferred by adults and adolescents, respectively. Based on previous research linking male sex, cigarette smoking, and the use of nicotine e-liquid to more frequent e-cigarette use [13], these variables were included as covariates in each model.

## Results

### Unadjusted differences between the adult and adolescent samples

Compared to adolescents, adults were more likely to be cigarette smokers, more likely to use nicotine e-liquid, and reported using e-cigarettes more frequently. Compared to adolescents, a larger percentage of adult e-cigarette users preferred tobacco, menthol, mint, coffee, and spice flavor e-liquids. Adults also preferred a greater total number of e-liquid flavors than did adolescents. Compared to adults, more adolescents preferred fruit, alcohol, and “other” flavored e-liquids or reported not knowing what their preferred flavor was. See Table 1 for complete chi-square and t-test results.

**Table 1:** Differences between the adult and adolescent samples on central study variables.

	Chi-Square Analyses		
	Adults	Adolescents	χ^2^
Males	53.7%	56.1%	0.52
Cigarette Smokers	51.2%	42.2%	7.72**
Nicotine E-liquid Users	82.9%	67.9%	29.80***
E-liquid Flavor Preferences			
*Tobacco*	32.0%	4.8%	105.60***
*Menthol*	27.6%	9.6%	47.47***
*Mint*	27.6%	9.1%	50.54***
*Fruit*	40.0%	52.3%	14.43***
*Coffee*	16.6%	6.8%	20.52***
*Vanilla*	11.5%	11.4%	0.01
*Candy/Dessert*	16.9%	16.2%	0.11
*Spice*	12.2%	3.5%	22.36***
*Alcohol*	6.3%	9.8%	4.26*
*Other*	0.3%	2.0%	6.67*
*I don't know*	0.0%	15.4%	96.88***
	Independent Samples T-tests		
	Adults	Adolescents	*t*
	*M* [*SD*]	*M* [*SD*]	
E-cigarette Use Frequency	15.56 [12.48]	9.98 [10.52]	7.57***
Total Flavors Preferred	1.91 [1.22]	1.26 [1.30]	8.08***

Note

* p < .05

** p < .01

*** p < .001

### Flavor preferences and e-cigarette use frequency in adults

Within the adjusted model examining adults’ preferences for the 10 e-liquid flavors as predictors of e-cigarette frequency, preferences for specific flavors were not significantly associated with e-cigarette use. Similarly, the total number of preferred flavors was not significantly associated with the number of days of e-cigarette use in the past month. See Table 2 for the complete results including model covariates.

**Table 2:** Predictors of e-cigarette use frequency in adults and adolescents.

	**Individual Flavor Preferences Predicting E-cigarette Use Frequency**					
	**ADULTS**			**ADOLESCENTS**		
	**R**^**2**^ **(Total Model) = 0.05**			**R**^**2**^ **(Total Model) = 0.17**		
**Independent Variables**	**B**	**Std. Error**	**η**_**p**_^**2**^	**B**	**Std. Error**	**η**_**p**_^**2**^
Males	-0.06	1.04	.00	2.86	1.01	.02**
Age	0.09	0.05	.01	0.22	0.43	.00
Cigarette Smokers	1.28	1.05	.00	4.73	1.11	.05***
Nicotine E-liquid Users	4.79	1.38	.02**	2.30	1.12	.01*
E-liquid Flavor Preferences						
*Tobacco*	-1.03	1.22	.00	-3.68	2.47	.01
*Menthol*	1.31	1.15	.00	0.04	1.86	.00
*Mint*	-0.95	1.18	.00	-0.01	1.77	.00
*Fruit*	-0.01	1.17	.00	2.98	1.01	.02**
*Coffee*	-2.42	1.39	.01	1.51	2.13	.00
*Vanilla*	2.09	1.77	.00	-0.69	1.72	.00
*Candy/Dessert*	2.14	1.59	.01	3.80	1.41	.02**
*Spices*	1.75	1.59	.00	0.96	2.90	.00
*Alcohol*	-2.77	2.15	.00	5.42	1.76	.02**
*Other*	15.30	8.84	.01	0.94	3.52	.00
* *						
	**Total Flavors Preferred Predicting E-cigarette Use Frequency**					
** **	**R**^**2**^ **(Total Model) = 0.03**			**R**^**2**^ **(Total Model) = 0.14**		
Males	-0.23	1.03	.00	2.76	1.01	.02**
Age	0.07	0.05	.00	0.15	0.43	.00
Cigarette Smokers	0.67	1.04	.00	4.30	1.08	.04***
Nicotine E-liquid Users	4.92	1.37	.02***	2.55	1.12	.01*
Total Flavors Preferred	0.26	0.42	.00	1.60	0.39	.04***

Note.

* *p* < .05

** *p* < .01

*** *p* < .001 E-cigarette frequency was defined as the number of days of e-cigarette use in the past 30-days

### Flavor preferences and e-cigarette use frequency in adolescents

In the adjusted model examining each of the 10 preferred flavors as predictors of e-cigarette use, adolescents who preferred to use fruit (52.3% of the sample; η_p_^2^ = 0.02, *p* = .003), dessert (16.2% of the sample; η_p_^2^ = 0.02, *p* = .007), and/or alcohol flavored e-liquids (9.8% of the sample; η_p_^2^ = 0.02, *p* = .002) reported using e-cigarettes more frequently. Additionally, the total number of e-cigarette flavors preferred was associated with e-cigarette frequency, such that preferring to use a greater number of e-cigarette flavors was associated with using e-cigarette on more days in the past month (η_p_^2^ = 0.04, *p* < .001). See Table 2 for complete results including model covariates.

Note that a separate model also was run in which the response “I don’t know” for flavor preference was included in the model as a predictor of e-cigarette use frequency. Given that the pattern of findings mirrored those presented above for adolescents and that no adults responded “I don’t know,” we present only the model in which the 10 preferred flavors were included to maintain consistency across the adult and adolescent samples.

## Discussion

The current study examined adolescent and adult e-cigarette users’ preferences for e-cigarette flavors and was the first to evaluate whether the flavor preferences of each group were associated with the number of days of e-cigarette use in the past month. Among adults, the most commonly preferred flavors were fruit (40.0%), tobacco (32.0%) and menthol/mint (27.6%), which mirrors previous research indicating the popularity of these flavors among adults [7], [16]-[17]. Among adolescents, the most commonly preferred flavors included fruit (52.3%), candy/dessert (16.2%), and vanilla (11.4%).

When comparing adults’ and adolescents’ preferences for specific flavors, on average, adults were more likely than adolescents to prefer flavors that may be perceived as non-sweet (e.g., tobacco, menthol, coffee, spice). It is not immediately evident why adults may be more likely to prefer non-sweet flavors like spices. However, adults may disproportionately prefer coffee flavors because they are more likely than adolescents, especially younger adolescents, to drink coffee [18]. Further, although it was not possible to test directly in the current study due to the lack of a specific question assessing motivations for e-cigarette use, adults may be more likely than adolescents to be using e-cigarettes as a substitute for cigarettes, and therefore prefer flavors that mimic traditional tobacco products like tobacco and menthol.

In contrast, adolescents were more likely than adults to prefer certain flavors that may be perceived as sweet (e.g., fruit, candy). These results are consistent with empirical findings indicating that sweet flavors (and smells) are disproportionately appealing to youth relative to adults [19]. Further, these results may have important implications for the regulation of flavor additives in e-cigarette products, given that preferences for specific sweet flavors predicted e-cigarette use exclusively among youth.

Regarding the total number of flavors preferred, adults preferred a greater total number of flavors than did adolescents. It is not immediately evident why adults preferred more flavors than did adolescents, but future research should investigate whether adults’ use of multiple flavors is linked to factors like e-cigarette experience (e.g., duration of e-cigarette use), device features (e.g., using customizable models that permit mixing multiple flavors together in the tank), economic ability to sample different e-liquid flavors, or the ability to purchase e-liquids legally from vape shops that often offer samples and sell a wide variety of flavors.

Adults used e-cigarettes more frequently, preferred a greater number of individual flavors, and preferred a greater total number of e-liquid flavors than did adolescents. However, neither adults’ preferences for individual flavors nor the total number of flavors they preferred was associated with the number of days of e-cigarette use in the past month. These findings suggest that the frequency of adults’ e-cigarette use was independent both of their flavor preferences and of the total number of flavors they prefer, and that adults who used multiple e-liquids flavors did not report using e-cigarettes more frequently than those who used fewer flavors. Instead, although the effects were modest, e-cigarette frequency among adults was related only to the use of e-cigarettes that contained nicotine.

In contrast, youths’ preferences for specific e-liquid flavors (i.e., fruit, dessert, and alcohol) and the total number of flavors they preferred were associated with more days of e-cigarette use. When considered in concert, the study findings indicate that preferences for specific, largely sweet flavors and the use of multiple flavors may play a greater role in adolescent e-cigarette use compared to adults. As noted in the introduction, past research indicating that flavored tobacco cigarettes were disproportionately appealing to youth led to the ultimate banning of flavored cigarettes (with the exception of menthol) by the FDA [4]. Given that the FDA’s regulatory authority now extends to e-cigarette products [20], the current findings may inform efforts to regulate e-liquid flavor additives. For example, although the availability of multiple e-cigarette flavors may have a positive benefit for adult smokers who are using e-cigarettes as a substitute for cigarettes, the availability of sweet flavors (e.g., fruit, candy) may contribute to youth e-cigarette use. Although it currently is illegal to sell e-cigarettes to individuals under the age of 18 years in the United States [20], future research is nonetheless needed to evaluate the effect of permitting certain flavors while limiting others on adult and adolescent e-cigarette use.

The current findings should be considered in light of several limitations. First, the study relied on self-report data, which are limited by participants’ willingness and ability to report honestly and accurately. Second, the cross-sectional design of the study also limits directional conclusions that can be drawn from the findings; future longitudinal work is needed to examine temporal relationships between e-liquid flavor preferences and e-cigarette use. Third, the generalizability of the findings may be limited by the fact that the adolescent sample comprised only high school students in Connecticut and the adult sample comprised MTurk workers who use e-cigarettes. Although both samples answered comparable questions, future research using data from national samples is needed. Fourth, we assessed preferences for broad e-liquid categories (e.g., fruit, desserts). As such, it was not possible to account for how many different types of e-liquids an individual may be using within a category. For example, an individual could regularly use five different types of fruit flavor e-liquids (e.g., cherry, blueberry, apple, strawberry, and watermelon), but this was only counted as a preference for one category in the current study. Future research is needed to determine relationships between e-cigarette use and the total number of e-liquids used (both within and across categories). Fifth, subsets of adolescents reported either not knowing what e-liquid flavor category they preferred or did not indicate a preference for any of the flavor categories included in the study. Future research is needed to explore these unique cases in greater depth. It is possible that adolescents who reported not knowing which flavor they prefer or who did not indicate a preference may have been unable to identify a preference as the result of their relative inexperience with e-cigarettes. For example, some youth may have been sharing others’ e-cigarettes and be unaware of the specific flavor they used because they did not purchase the e-liquid directly. Further, uncertainty about flavors may be linked to the fact that some e-juice flavors have a name that does not clearly correspond to one of the specific flavor categories assessed in the study (e.g., Boss Sauce Reserve; Bird Brain; Unicorn Milk). It also is possible that some adolescents simply could not make up their minds or truly were indifferent about the flavors. Sixth, it was not possible to determine exactly how participants who reported preferring multiple e-liquid flavors were using these flavors. For example, participants may have been purchasing multiple disposable e-cigarettes each with a different flavor, mixing multiple preferred flavors together in a tank, or refilling a single tank with a new preferred e-liquid flavor once the previous preferred flavor ran out. Future research should assess the different ways in which multiple flavors may be used, as different approaches may differentially relate to e-cigarette use and could have contributed to the differences observed between adolescents and adults in the current study. Seventh, our assessment of the use of e-liquids containing nicotine was limited in the current study to nicotine use versus no nicotine use. Given the addictive nature of nicotine [21], using higher nicotine concentrations may prompt more frequent use. Therefore, future research examining the impact of using varying concentrations of e-liquid on adolescent and adult e-cigarette use is warranted. Eighth, our assessment of cigarette smoking also was limited in the current study (i.e., any use in the past 30 days). Future studies should assess the impact of cigarette smoking on e-cigarette use behaviors using more nuanced smoking measures (e.g., duration of use, heaviness of smoking). Finally, the adolescent data were collected in 2014 prior to the enactment of 2015 Connecticut legislation [22] and 2016 national FDA legislation [20] banning the sales of e-cigarettes to minors. In light of recent evidence from the 2016 National Youth Tobacco Survey demonstrating that past-month e-cigarette use by youth declined for the first time since e-cigarette use was assessed [23], it is possible that laws banning the sales of e-cigarettes to minors may have an impact on the replicability of the findings reported in the current study. Thus, future research is needed to examine the extent to which the reported pattern of results may be influenced by increased regulatory efforts.

Despite the limitations, the current study provides preliminary evidence that specific flavor preferences and the use of multiple flavors is associated with more frequent e-cigarette use among youth and that a similar pattern of results was not observed within an adult sample. The findings may be used to spur addition research that contributes to regulatory action. The availability of a wide variety of e-liquid flavors may ease the transition to e-cigarette use and contribute to trajectories of continued e-cigarette use (and potential nicotine exposure) given that youth may be especially attracted to certain flavors and the novelty of trying new flavors. On the other hand, the flavor preferences of adults, who may prefer different flavors than adolescents because they are using e-cigarettes for different reasons (e.g., to replace cigarette smoking), may not relate to e-cigarette use. In sum, to enhance the overall net population benefit of e-cigarette product availability, regulatory efforts ultimately may consider restricting specific flavors. For example, it may be important to maintain e-cigarette flavors that appeal to adults, if evidence emerges that these flavors aid in smoking cessation. However, it also may be important to restrict certain e-cigarette flavors that uniquely are associated with more frequent e-cigarette use among youth.

## Supporting information

S1 Data (SAV)
