# Correction: Preferring more e-cigarette flavors is associated with e-cigarette use frequency among adolescents but not adults

**DOI:** 10.1371/journal.pone.0204349

**Published:** 2018-09-13

**Authors:** Meghan E. Morean, Ellyn R. Butler, Krysten W. Bold, Grace Kong, Deepa R. Camenga, Dana A. Cavallo, Patricia Simon, Stephanie S. O’Malley, Suchitra Krishnan-Sarin

There are errors in the Funding statement. The correct funding information is as follows: Research reported in this publication was supported by the National Institutes of Health/NIDA and FDA Center for Tobacco Products (CTP) (P50DA036151; Yale Tobacco Center of Regulatory Science, TCORS); the National Institute on Drug Abuse (T32DA019426); and the National Center on Addiction and Substance Abuse at Columbia University (to G.K.). The content is solely the responsibility of the authors and does not necessarily represent the official views of the funding agencies, including the NIH or the Food and Drug Administration. The funders had no role in study design, data collection and analysis, decision to publish, or preparation of the manuscript.
